# Clustered precursors in bone marrow sections predict early relapse in patients with acute myeloid leukemia within hematologic remission

**DOI:** 10.1186/1479-5876-12-18

**Published:** 2014-01-22

**Authors:** Yehua Yu, Zhentian Wu, Jing Zhang, Yuanmei Zhai, Yinghua Yuan, Sihong Liu, Hui Wang, Jun Shi

**Affiliations:** 1 Department of Hematology, Shanghai Jiao Tong University Affiliated Sixth People’s Hospital, Shanghai, China

**Keywords:** Bone marrow, Biopsy, Relapse, Acute myeloid leukemia

## Abstract

**Background:**

Bone marrow (BM) aspiration is largely used for relapse assessment in acute myeloid leukemia (AML). It remains unclear what roles that BM trephine biopsy plays on relapse assessment.

**Methods:**

Bone marrow (BM) sections during complete remission (CR) from 60 acute myeloid leukemia (AML) patients were retrospectively analyzed. Computer image processing technology was performed for detection of the distance between precursors and endosteum, and density of precursors was also calculated under light microscopic image. Immunohistochemistry was used to identify the immunophenotype of clustered precursors.

**Results:**

Except for single and double precursors, there existed clustered precursors of 3-5 cells during CR. Here, we demonstrated that clustered precursors, but not single and double precursors, were useful in risk factor of relapse. Area under the receiving operator curve (ROC) was of 0.007 (CI 95%, from 0.572 to 0.851). Using a standard cut-off value of >4.0/mm^2^ for cluster density, early relapse was detected with a sensitivity of 51.5% and a specificity of 85.7%.

Multivariate Cox regression analysis revealed that clustered precursor is an independent risk factor for early relapse (Adjusted HR: 0.325, 95% CI: 0.156-0.679, p = 0.003).

**Conclusions:**

Cumulatively, clustered precursors in BM sections during CR may serve as an independent risk factor of early relapse and poor outcome for AML patients in cluster density > 4.0/mm^2^ in sections. Early aggressive interventions are needed to prevent hematologic relapse.

## Introduction

In leukemia, the leading cause of treatment failure is disease relapse. Salvage treatments for relapsed patients frequently lead to dismal outcomes including low levels of complete remission (CR) and short overall survival (OS) times [1-4]. Hitherto, the traditional approach to assess treatment response and follow-up has relied on counting blast cells in bone marrow (BM) smears. BM smears, however, may occasionally be diluted by sinusoidal blood and subsequently cannot provide reliable information on treatment response and fail to judge early relapse [5]. It may even be inferior to the BM imprint found in trephine biopsy for the evaluation of cellularity, at least according to a recent report [6]. In addition, BM aspiration only provides information on cellularity and lacks other information such as cellular localization and microenvironmental structure. BM biopsy sections might compensate for these shortcomings [7].

Similar to the observations made for cell numbers, cell localization might also provide important information applicable to disease diagnosis and perhaps prognosis assessment. In normal human subjects, precursors are rare and are found localized near the endosteum, and consist of 1-2 cells. In some cases of myelodysplasia syndrome (MDS), immature precursors might be located in the intertrabecular region and occasionally aggregate as clusters which are of 3 ~ 5 cells, such clusters were initially defined as abnormal localization of immature precursors (ALIP) by Tricot [8]. ALIP prominently presented in high-risk MDS patients and the delay prior to transforming to acute myeloid leukemia (AML) was much shorter as compared with ALIP-negative MDS patients [8-10]. It thus suggested that abnormal localization of precursors could assist in the assessment of patient prognosis.

In the present study, we found during CR of AML patients, there existed clustered precursors consisting of 3 to 5 cells in the intertrabecular region. Since they were morphologically and anatomically analogous to ALIP in MDS, we defined them as ALIP-like clusters. The precise information that histological data provides during CR is largely unknown. Here, we extended our investigations to explore a histological relapse indicator by comparing the frequency of ALIP-like clusters between relapse and no-relapse cases.

## Materials and methods

### Patients

Between December 2004 and February 2013, 115 patients with de novo AML were admitted to the Hematology Department of Shanghai Sixth People’s Hospital, China. Patients were diagnosed according to the FAB classification. CR and relapse were diagnosed according to Cheson et al. [11]. Those patients who did not receive stem cell transplantation were enrolled in this study. Cases were excluded from the study if they: (a) lacked complete clinical data; (b) presented with other diseases that might impact on chemotherapy; or (c) died because of other disease but not of leukemia. With those exclusion criteria, 60 patients were enrolled in this study. For induction therapies, AML patients were treated with one anthracycline agent (daunorubicin, idarubicin, epirubicin or mitoxantrone at a daily dosage of (40–60) mg/m^2^, 7-8 mg/m^2^, (60–90) or (8–10) mg/m^2^, respectively) for 3 days and cytarabine at (100–150) mg/m^2^ daily for 7 days. For consolidation therapies, AML patients received up to 6 cycles of induction therapies including one alternative anthracycline with similar dosage mentioned above. All trephine biopsies and administrations were performed after having obtained informed consent. This study was approved by the institutional Ethics Committees of our hospital and conducted in accordance with the ethical guidelines of the Declaration of Helsinki. First check-up post induction was assessed by bone marrow biopsy on day 21–28 post induction. Concerning the physical condition of the patients, the biopsies might be retarded until peripheral blood recovery. During CR, BM biopsies were routinely performed before every consolidation therapies. After consolidation therapies, bone marrow was surveilled by biopsies every 3 months in the first year and every 6 months in the second year. BM sections and smears from the patients were assayed under paired analysis from the time of diagnosis to the follow-up. Cases in CR were divided into relapse and no-relapse groups according to patients relapsed or not during the follow-up. The pathologists who examined the BM samples were not participant of this study and were innocent about the group situation. Clinical outcome was assessed by relapse-free survival (RFS) and overall survival (OS).

### Determination of the distance from precursors to endosteum and precursor density on BM sections

BM biopsy samples were collected from AML patients during CR and fixed in Bouin fixative. Dehydration was performed by exposing specimens to ascending concentrations of ethanol. All samples were embedded in Hemapun865 plastic. The component of the plastic and the detailed embedding technique had been previously described [12]. BM sections of 3 μm thickness were stained with haematoxilin-giemsa-acid fuchsin (HGF) for visualization of immature precursor cells. Specimens were observed by using of optical microscope imaging system (Olympus, Tokyo, Japan). Precursors were sub-grouped into single, double and clustered cells that consisted of ≥ 3 precursors. For every case, sections were observed randomly by 10 fields of view with × 400 magnification. For detection of the precise distance between precursors and endosteum, computer image processing technology was performed [13]. The distance detected by computer as pixel was transferred into μm by the algorithm of 1 pixel = 0.2 μm. For detection of the density of precursors found in BM sections, a hemocytometer was used, wherein the 16 smallest square lattices were set as 1 mm^2^.

### Immunohistochemical (IHC) staining

To study the cellularity of ALIP-like clusters, IHC staining was performed. The formalin-fixed, EDTA (PH 7.4) decalcificated and paraffin-embedded bone marrow tissues were cut at a section thickness of 3 μm. The staining procedure was done in accord with the instructions provided with an UltraVision Quanto Detection System HRP DAB kit’s (Thermo Scientific, TL-060-QHD, CA, USA). In brief, sections were deparaffinized in xylene and rehydrated by exposure of the specimens to graded ethanol. Antigen retrieval was performed by sodium citrate buffer with pH 6.0 at 90°C for 15 min. Mouse anti-human CD34 Class II monoclonal antibody (1:100 diluted, M7165,Dako, Glostrup, Denmark), rabbit anti-human CD117 polyclonal antibody (1:200 diluted, A4502, Dako, CA, USA), and mouse anti-human myeloperoxidase (MPO) antibody (1:8 diluted, R-0405, Changdao, Shanghai, China) were incubated at 4°C over night. Horseradish peroxidase (HRP) labeled secondary antibody and the substrate diaminiobenzidine (DAB) were used according to the kit’s instructions. Then sections were viewed under the light microscope (Olympus, Tokyo, Japan).

### Statistical analysis

Mann–Whitney U test was applied to compare the density of precursors between the relapse and no-relapse cases and their distance to endosteum in every cell group. Chi-square test and Fisher’s exact test were used for categorical data. A receiver operating characteristic curve (ROC) analysis was performed and the corresponding area under the curve (AUC) was calculated to determine the value of the precursor density in predicting a recurrence. Survival curves were constructed using the Kaplan–Meier method and the log-rank test was used to test the difference in RFS and OS between cases with different densities of ALIP-like clusters. RFS was defined as the time between diagnosis and the onset of the first relapse, and OS was defined as the time between diagnosis and the occurrence of death or when lost to follow-up. Cox proportional hazard regression was used to model the RFS. The data were described as mean ± standard deviation (SD), *P* values ≤0.05 were considered statistically significant. All statistical analyses were conducted by using the SPSS 18.0 software program (Statistical Package for Social Science, SPSS Inc. Chicago, IL., USA).

## Results

### Characteristics of the patients

Ages of patients in the relapse group and no-relapse group were (51.85 ± 17.96) years and (49.86 ± 16.96) years respectively. The ratios of male/female in the two groups were 24/15 and 10/11 respectively (Table 1). There were no statistical differences in age, sex and FAB subtypes between the two groups.

**Table 1:** Patient demographics and clinical characteristics

**Characteristic**	**No-relapse group (n = 21)**	**Relapse group (n = 39)**	***P *** **value**
Age (years)			0.678
Mean ± SD	49.86 ± 16.96	51.85 ± 17.96	
Range	20-80	16-77	
Sex (male/female)	10/11	24/15	0.414
FAB subtype	-	-	0.885
M1	0	4	
M2	8	14	
M3	5	8	
M4	4	6	
M5	2	4	
M6	1	1	
M7	1	2	

FAB: French-American-British Classification.

### Cellularity and localization of precursors in BM histologcal sections during CR of AML

**Figure 1:**
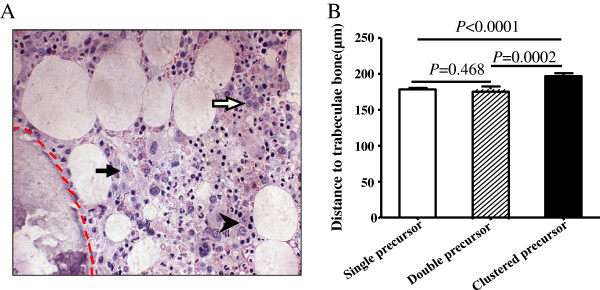
**Distances to the endosteum among different groups of cells. (A)** Representative histological section from an AML patient in CR. Solid arrows indicated single precursor, arrow head indicated double precursors, and hollow arrow indicated clustered precursor. Dashed red line highlighted the endosteal surface. Staining of sections was done by haematoxilin-giemsa acid fuchsin (HGF), and observed at × 400 magnification. **(B)** Single and double precursors were located near the endosteum and there was no statistical difference between the two groups. By contrast, the clustered precursors were located further from endosteum than single and double precursors.

During CR, there exited single, double and clustered precursors in BM sections (Figure 1A) .The exact distances from the center of the precursors or clusters to the endosteum were detected by computer processing image. Clustered precursors were located further than single precursors and double precursors (*P* < 0.0001 and *P* = 0.0002, respectively, Figure 1B) and consequently in the intertrabecular region. The distance to the endosteum between single and double precursors showed no significant difference (*P* = 0.468, Figure 1B).

### Density of ALIP-like clusters during CR is associated with relapse risk

**Figure 2:**
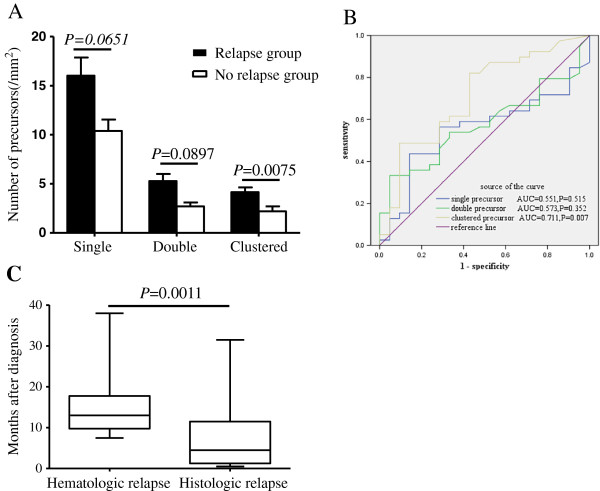
**ALIP-like clusters during CR in AML patients were considered the best indicator of early relapse among the three precursor group. (A)** Densities of single, double, and clustered precursors were higher in the pre-relapse cases. **(B)** showing ROC analysis for the prediction of relapse from single, double and clustered precursors. This analysis showed that ALIP-like clusters mostly favored the prediction of relapse with AUC = 0.711. AUC: Area under curve. **(C)** The median time from histologic relapse to overt hematologic relapse.

**Figure 3:**
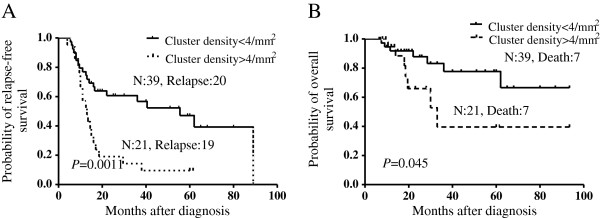
**Exceeding ALIP-like cluster density (>4/mm**^**2**^**) indicated poorer survival.** Contrast to cases with <4/mm^2^ of ALIP-like cluster density, cases with exceeding ALIP-like cluster density (>4/mm^2^) carried poorer outcome in both RFS **(A)** and OS **(B)**.

As shown in Figure 2a, the density of single and double precursors in relapse group increased in trace level but indicated no statistical significance when compared with no-relapse group (*P* = 0.0651 and 0.0897 respectively). However, clustered precursors in relapse patients were significant higher than those in no-relapse patients (*P* = 0.0075). In ROC analysis of recurrent prediction, single, double and clustered precursors resulted in an AUC of 0.551, 0.573, and 0.711, respectively (Figure 2B) and with *P* value 0.515, 0.352 and 0.007, respectively. This observation suggested that the strongest predictor for leukemic relapse was clustered precursors, and the threshold value of the cluster density was 4.0/mm^2^ to achieve the maximum sum of both sensitivity and specificity (Table 2). Subsequently, in order to evaluate the sensitivity of histological appearance in relapse prediction, time-point of histological relapse (cluster density > 4.0/mm^2^) and hematologic relapse were compared. As a result, the median time from histologic relapse to hematologic relapse was 8 months (Figure 2C). Additionally,the probability of RFS and OS were compared between cases with cluster density > 4.0/mm^2^ and those less than 4.0/mm^2^. As shown in Figure 3A-B, there was a poorer prognosis in RFS (*P* = 0.0011) and OS (*P* = 0.045) for cases with cluster density > 4.0/mm^2^.

**Table 2:** Sensitivity and specificity of ALIP-like cluster density in identifying relapse risk according to ROC curve analysis

**Cut-off clusters**	**Sensitivity**	**Specificity**
1.0000	.848	.357
2.0000	.727	.500
3.0000	.636	.643
4.0000*	.515	.857
5.0000	.364	.857
6.0000	.182	.857
7.0000	.121	.857
8.0000	.091	.929
9.0000	.061	.929
10.0000	.061	1.000

*The threshold value of cluster density in BM sections for predicting relapse.

### Prognostic assessment of known clinical parameters

In the univariate and multivariate Cox regression model, only the factor of cluster density > 4.0/mm^2^ was associated with higher relapse risk (*P* = 0.002 and 0.003, respectively Table 3). Other candidate prognostic factors including bone marrow precursors, peripheral white blood cells and age, were not associated with higher relapse risk in both univariate and multivariate model (Table 3).

**Table 3:** Cox regression analysis of RFS in AML patients during CR

**Variables**	**Univariate analysis**			**Multivariate analysis**		
	**HR**	**95% CI**	**P value**	**Adjusted HR**	**95% CI**	**P value**
ALIP-like cluster^*^	0.353	0.183-0.680	0.002	0.325	0.156-0.679	0.003
BMP	1.381	0.408-4.670	0.604	2.358	0.598-9.290	0.220
PWBC	1.011	1.000-1.023	0.051	1.008	0.997-1.020	0.164
Age	1.008	0.990-1.026	0.408	1.001	0.980-1.022	0.944

*Abbreviations*: *BMP* Bone marrow precursors, *HR* hazard ratio, *CI* confidence interval, *PWBC* peripheral white blood cell.

^*^ALIP-like cluster density > 4/mm^2^ versus < 4/mm^2^.

### ALIP-like clusters were composed of immature myeloid precursors

**Figure 4:**
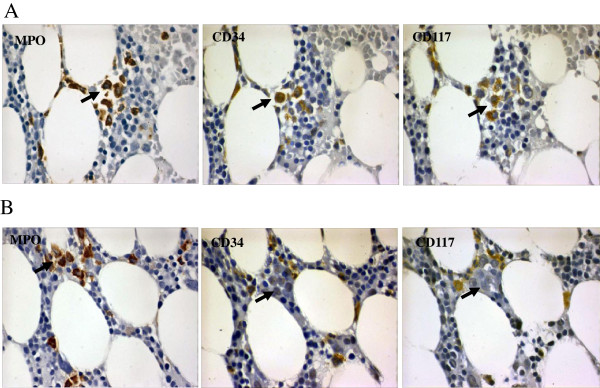
**ALIP-like clusters are of heterogeneous myeloid precursors.** All ALIP-like clusters expressed MPO. Some clusters expressed both CD34 and CD117 **(A)**. However, other clusters expressed neither CD34 nor CD117 **(B)**. Sections were observed at × 1000 magnification. Arrows indicated ALIP-like clusters.

To clarify whether ALIP-like cluster were composed of immature myeloid precursors, assay of staining for MPO was selected to confirm the myeloid lineage, and CD34 and CD117 were chosen for staining immature precursors. As a result, all ALIP-like clusters were MPO positive. However, the expression of CD34 and CD117 was shown to be variable. As shown in Figure 4A, precursors in some ALIP-like clusters expressed both CD34 and CD117. In the other populations, however, they expressed neither (Figure 4B).

## Discussion

Since single and double precursors are observed in healthy human BM sections and are located near endosteum, we found it intriguing to know how the precursors and especially ALIP-like clusters were located in AML in CR. In this setting, we detected the distances between precursors and the endosteum in BM sections by computer image processing technology. As a result, single and double precursors in CR of AML were also observed near the endosteum. ALIP-like clusters, however, were located farther from the endosteum. Furthermore, IHC assays showed that these clusters were of myeloid lineage, and strongly supported the sense that the clusters were ALIP-like.

We hypothesized that similar with the prognostic value of ALIP in MDS, ALIP-like cluster in CR of AML patients should also be considered as a prognostic factor. Indeed, the frequency of ALIP-like cluster was higher in relapse group than in no-relapse group. However, it was interesting that frequencies of single and double precursors were not statistically higher in relapse cases than in no-relapse cases. Since clustered precursors are only observed in BM sections from malignant diseases (MDS and CR of AML) or some benign disease with impaired BM, such as BM necrosis and stromal alterations in systemic lupus erythematosus patients [14], but not in normal BM sections, we speculate that in CR of AML, clustered precursors, but not single and double precursors, are more characteristic for malignant hematopoiesis in BM sections, and it is thus considered that only ALIP-like cluster could be acted as relapse predictor. With ROC analysis, it testified that ALIP-like clusters were most favorable for relapse prediction than single and double precursors. We subsequently established that cluster density of 4.0/mm^2^ as the most optimal threshold value with the most predictive power and it might predict relapse with 8 months earlier than hematologic relapse. Survival analysis further verified that cases with histologic relapse carried a poorer clinical outcome in both RFS and OS. To determine whether ALIP-like clusters are an independent predict marker at high risk of early relapse, Cox proportional hazards model was performed. In this model, it indicated that ALIP-like cluster was the independent risk factor for assessing early relapse. Here, we propose that in BM sections, cluster density more than 4/mm^2^ during hematological remission to be referred to as histologic relapse, and more aggressive adjuvant treatment is needed to prevent hematologic relapse.

Our observations showed that single and double precursors were located near the endosteum, while ALIP-like clusters were in intertrabecular region. This is analogous with a recent study that showed that single normal hematopoietic stem cells (HSC) were located close to the endosteum. However, more mature precursors were located farther away from the endosteum and expanded to clusters [15]. Likewise, chemo-resistant leukemic stem cells (LSC) were prominently located near the endosteum after chemotherapy in an AML mice model [16]. In this setting, we hypothesized that ALIP-like clusters evolved from LSC harboring at sites near the endosteum, while under conditions of administration retrieval, LSC might exhibit differentiation under the influence of some cytokine regulators such as vascular endothelial cell growth factor [17-19] and insulin-like growth factor [20]. Under those situations, the progeny cells migrate toward the intertrabecular region [21]. In the context of this hypothesis, ALIP-like clusters might be heterogeneous and hierarchical with more mature population located in intertrabecular region. Contrarily, more immature population might be located closer to the endostreum. IHC staining indicated that the progeny cells shared variable immuno-phenotype, wherein some sub-populations were CD34/CD117 positive, while the others are negative. These observations supported that analogous with LSC, the daughter cells were also heterogeneous and hierarchical in behavior.

In this current study, we propose that at a value of greater than 4.0/mm^2^ of ALIP-like cluster density in BM sections within hematologic remission is a strong independent prognostic factor for AML after achieving a complete remission, and consider it as an indication of histologic relapse and early interventions might be considered as a pre-requisite in histological relapse to improve eventual clinical outcomes. Here, we suggest BM biopsies should be considered while other minimal residual disease measurements such as reverse transcription-polymerase chain reaction (RT-PCR), flow cytometer, etc. are not available. Nonetheless, there are some limitations in this study. Firstly, cytogenetic and molecular marker assays are not routinely performed at this hospital, comparison and combination with other MRD measurements such as RT-PCR, flow cytometer, etc. [22,23] are lacking. Secondly, we could not exclude the influence of cytogenetic subtypes on relapse and clinical outcome. A further prospective blinded study is required to assess the clinical significance of the cut-off values in relapse prediction.

## Conclusion

Taken together, in this retrospective study, we found during CR of AML patients, except for single and double precursors, there existed clustered precursors of 3 ~ 5 cells. The clustered precursors are morphologically and anatomically analogous to ALIP in MDS. Similar with the prognostic value of ALIP in MDS, these ALIP-like clusters in BM sections during CR of AML could also be used as an independent predictor of early relapse and poor outcome for AML patients. Finally, we proposed that while ALIP-like cluster density increased to over the threshold of 4.0/mm^2^, although in hematological response, urgent interference should be needed to prevent early recurrence and consequently improve the OS.

## Competing interests

The authors declare that they have no competing interests.

## Authors’ contributions

YY performed laboratory tests and collected patient data and analyzed the data. ZW analyzed the patient data and wrote the paper. JZ, YY, YZ, SL and HW performed the laboratory tests. JS provided patient data, analyzed the data and wrote the paper. All authors read and approved the final version of the submitted manuscript.
